# St. John's Hospital for Diseases of the Skin

**Published:** 1904-10-08

**Authors:** 


### EDITOR'S LETTER-BOX.

[Our Correspondents are reminded that prolixity is a great bar to publication, and that brevity of style and conciseness of statement greatly facilitate early insertion.]

### ST. JOHN'S HOSPITAL FOR DISEASES OF THE SKIN.

The Secretary, Mr. G. A. Arnaudin, informs us that during the rebuilding of the Out-Patient Department of St. John's Hospital for Diseases of the Skin in Leicester Square, the work of the hospital is being carried on at 49 Rupert Street, Coventry Street, W.

